# Effects of at‐risk drinking on central hemodynamics and aortic stiffness in midlife adults

**DOI:** 10.14814/phy2.70717

**Published:** 2026-01-11

**Authors:** Keng‐Yu Chang, Zhaoli Liu, Hitesh Nirmal, Brooks A. Hibner, Maysa Nashawati, John O. Kolade, Yeonwoo Kim, R. Matthew Brothers, Shane A. Phillips, Chueh‐Lung Hwang

**Affiliations:** ^1^ Department of Kinesiology University of Texas at Arlington Arlington Texas USA; ^2^ College of Nursing and Health Innovation University of Texas at Arlington Arlington Texas USA; ^3^ Department of Kinesiology and Nutrition University of Illinois at Chicago Chicago Illinois USA; ^4^ Department of Physical Therapy University of Illinois at Chicago Chicago Illinois USA

**Keywords:** alcohol drinking, arterial stiffness, blood pressure, cardiovascular disease risks, wave reflection

## Abstract

The purpose of this cross‐sectional study was to determine the effect of at‐risk drinking on central hemodynamics and aortic stiffness in midlife adults. A total of 38 midlife men and 41 postmenopausal women, aged 50‐64 and free of major clinical diseases, were included. Based on USAUDIT‐C scores derived from the U.S. Alcohol Use Disorder Identification Test, participants were classified as low‐risk drinkers (*n* = 50) or at‐risk drinkers (*n* = 29). Central blood pressure (BP), aortic wave reflection indices, as well as carotid‐to‐femoral pulse wave velocity (cfPWV; a measure of aortic stiffness) were measured. Regardless of sex (*p* = 0.11 for both), among participants free of antihypertensive medications (*n* = 51), at‐risk drinkers had higher central systolic (*p* = 0.002) and diastolic BP (*p* < 0.001) compared with low‐risk drinkers, while there was no between‐group difference in central BP among treated participants (*n* = 28; *p* ≥ 0.41). Among untreated participants, higher USAUDIT‐C scores remained independently associated with elevated systolic (*p* < 0.001) and diastolic BP (*p* = 0.003), after controlling for wave reflection indices and cfPWV. Regardless of antihypertensive medication use (*p* ≥ 0.25) and sex (*p* ≥ 0.10), no between‐group differences were observed in aortic wave reflection indices (*p* ≥ 0.18) and cfPWV (*p* = 0.16). These findings suggest that elevated central BP associated with at‐risk drinking is related to mechanisms other than enhanced aortic wave reflection or aortic stiffening.

## 1 INTRODUCTION

Hypertension impacts over half of midlife adults who are 50–64 years old (Martin et al., 2025) and is a major driving risk factor for cardiovascular disease (CVD). The 2025 American College of Cardiology (ACC) and American Heart Association (AHA) High Blood Pressure (BP) Guideline recommends either reducing or eliminating alcohol consumption for hypertension prevention and management (Jones et al., 2025). When alcohol is consumed, men should limit their intake to no more than two drinks per day and 14 drinks per week and women should limit their intake to no more than one drink per day and seven drinks per week (one drink contains 14 g of ethanol) (Dietary Guidelines for Americans, 2020; Piano et al., 2025). In addition, adults should avoid binge drinking, that is having five drinks or more per sitting for men and four drinks or more per sitting for women (Dietary Guidelines for Americans, 2020; Piano et al., 2025). However, alcohol remains the most widely used substance in the United States and at least one in five midlife adults consume alcohol over the recommended drinking limits (Grucza et al., 2018). These unhealthy alcohol use patterns are associated with several adverse CVD outcomes, including an increased risk of hypertension (Cecchini et al., 2024; Di Federico et al., 2023), and are usually referred as at‐risk drinking.

Growing evidence indicates that at‐risk drinking is associated with increased aortic stiffness (Hwang et al., 2022), which is a known contributor to increased risks of hypertension and CVD (Ben‐Shlomo et al., 2014; Vlachopoulos, Aznaouridis, & Stefanadis, 2010). Increased aortic stiffness can cause early return and/or increased magnitude of wave reflection from the periphery to the aorta. In young healthy adults, reflected waves usually return to the heart during diastole, which supports coronary perfusion. In contrast, early return of these waves during systole augments central systolic BP, increases left ventricle workload, and reduces coronary perfusion, eventually causing pathophysiological changes in the heart (Mitchell et al., 2004). Measures of central hemodynamics, including central BP and aortic wave reflection, not only reflect pressure load imposed on the heart and the brain (Hashimoto, 2017), but also CVD risks. While peripheral BP is commonly used to define hypertension and assess CVD risks, central hemodynamic measures have been shown to provide a similar (Vlachopoulos, Aznaouridis, O'Rourke, et al., 2010) or even better (Dong et al., 2020) prediction of CVD events compared to peripheral BP.

Although the pressor effect of alcohol use is well established, it is based on peripheral BP (Cecchini et al., 2024; Di Federico et al., 2023). Only a handful of studies have examined the effect of alcohol consumption on central BP and wave reflection in midlife adults, and the findings are contradictory (Basdeki et al., 2021; Mahmud & Feely, 2002; Sierksma et al., 2004). Sierksma et al. (2004) reported that in postmenopausal women, alcohol use was unrelated to both central systolic BP and aortic wave reflection. Basdeki et al. (2021) also reported that there was no association between alcohol consumption and central BP in women or men; however, in men, alcohol consumption of more than 30 g (~2 drinks) per day was associated with higher aortic wave reflection (Basdeki et al., 2021). Mahmud and Feely reported no difference in central BP and aortic wave reflection between women who consumed more than 10 drinks per week and those who drank less. However, men who consumed more than 15 drinks per week had higher central BP and enhanced aortic wave reflection compared with those who drank less (Mahmud & Feely, 2002). These discrepancies across studies may be related to sex differences, as well as differences in alcohol use assessment methods, drinking classifications, absence of thresholds relative to the current guidelines, and control for antihypertensive medication use. Thus, important gaps remain in understanding the effect of at‐risk alcohol consumption on central hemodynamics.

The purpose of this study was to examine the effects of at‐risk drinking on central hemodynamics, including central BP and aortic wave reflection, as well as aortic stiffness in midlife adults. We hypothesized that compared with midlife adults who drank within the daily and weekly recommended drinking limits (low‐risk drinkers, including abstainers), those who drank over the limits (at‐risk drinkers) would have higher central BP, enhanced wave reflection, and increased aortic stiffness. We also hypothesized that these effects would be influenced by sex and use of antihypertensive medications.

## 2 METHODS

### 2.1 Study design

This cross‐sectional study was phase 1 of an ongoing study examining the effect of alcohol use and alcohol abstinence on BP in midlife adults (NCT05522075) (Chang et al., 2025). All procedures were approved by the Institutional Review Boards of the University of Illinois at Chicago (IRB# 2020‐0390) and the University of Texas at Arlington (IRB#2022‐0189). Written informed consent was obtained from all research participants before any in‐laboratory measurements were performed. This study conformed to the Declaration of Helsinki.

### 2.2 Participants and drinking group classification

A total of 38 men and 41 women aged 50–64 years were included in this study (84% Whites and 87% non‐Hispanics). As ascertained by self‐report medical history, all participants were nonsmokers and free of any major clinical diseases, including CVD, diabetes, liver, and renal disease. All participants had office BP <160/100 mmHg, body mass index (BMI) <35 kg/m^2^, and fasting low‐density lipoprotein <190 mg/dL as confirmed by physical examination and fasting blood samples. None of the participants were on hormone replacement therapy ≥1 year prior to the study visit, and all women were postmenopausal based on self‐report cessation of menses ≥1 year. None of the participants reported regular aerobic exercise (≥30 min/time of moderate intensity aerobic exercise, 3 times/week). Participants who were currently taking antihypertensive medications ≥2 months were also included. Details regarding participant recruitment, screening procedures, as well as inclusion and exclusion criteria were published elsewhere (Chang et al., 2025).

To classify drinking groups, participants completed the first three questions from the U.S. Alcohol Use Disorder Identification Test (USAUDIT‐C) regarding the amount of weekly consumption and occasions of binge drinking over the past year and the USAUDIT‐C score was calculated (range 0–18) (Chang et al., 2025). Similar to other studies (Babor et al., 2006; Esselink et al., 2023; Verhoog et al., 2020), men with scores <8 and women with scores <7 were categorized as low‐risk drinkers, and men with scores ≥8 and women with scores ≥7 were categorized as at‐risk drinkers. At‐risk drinkers were defined as those who consumed alcoholic beverages above the NIAAA low‐risk daily and weekly limits, which are no more than four drinks/day and no more than 14 drinks/week for men and no more than three drinks/day and no more than seven drinks/week for women (one standard drink contains 14 g of ethanol). To understand alcohol consumption over the past 2–4 weeks, an alcohol biomarker, phosphatidylethanol (PEth), was measured in dried blood spots by high performance liquid chromatography with mass spectrometry (U.S. Drug Testing Lab; Des Plaines, IL), as published previously (Chang et al., 2025). This biomarker is only formed when alcohol is consumed and can be detectable up to 2–4 weeks after alcohol consumption. To understand alcohol consumption over the past 2 weeks, participants completed a 14‐day Timeline Follow‐Back Calendar, and average drinks per week, average drinking days per week, and binge drinking days per week were calculated.

To understand participant characteristics, including physical activity, diet, sleep, and mental health, participants completed the 4‐day physical activity monitoring (to determine time spent on moderate‐to‐vigorous intensity physical activity and daily steps), the Dietary Habit Survey, the Pittsburgh Sleep Quality Index, the eight‐item Personal Health Questionnaire Depression Scale, and the State–Trait Anxiety Inventory. Details in these measurements were published previously (Chang et al., 2025).

### 2.3 Measurements

Measurements were performed in the morning after a ≥12‐h fast, ≥12‐h abstinence from caffeine and exercise, and ≥24‐h abstinence from alcohol. Prescribed medications, including antihypertensive medications, were continued. Blood pressure, aortic wave reflection, and aortic stiffness were assessed after a supine rest ≥10 min in a quiet, semi‐darkened, and temperature‐controlled room.

### 2.4 Central blood pressure and wave reflection

As we published previously (Hwang et al., 2014, 2021), central BP and wave reflection characteristics were assessed noninvasively using the SphygmoCor XCEL system (SphygmoCor; AtCor Medical, Australia). Briefly, peripheral BP and pressure waveforms were obtained over the left brachial artery. Central aortic pressure waveforms were generated using a general transfer function of the acquired peripheral waves to determine central systolic BP, diastolic BP, and pulse pressure (PP). Using pulse wave analysis, augmentation pressure (AP) was obtained, and augmentation index (AIx) was calculated as the ratio of the AP to the central PP. Using wave separation analysis, the height of forward (Pf) and reflected (Pb) central pressure waves was obtained, and the reflection magnitude (RM) was calculated as the ratio of Pb to Pf amplitude.

### 2.5 Aortic stiffness

As we described previously (Hwang et al., 2014, 2021), aortic arterial stiffness was measured as carotid‐to‐femoral pulse wave velocity (cfPWV) via simultaneous acquisition of pressure waves at the left carotid and femoral artery using the SphygmoCor XCEL system (SphygmoCor; AtCor Medical, Australia). Following the manufacturer's instructions, cfPWV was calculated as the ratio of the distance to pulse transit time between arterial sites. Given that cfPWV is BP dependent, cfPWV was normalized to brachial mean arterial pressure (MAP).

### 2.6 Statistical analysis

Statistical analyses were performed by using the IBM SPSS Statistics (Essentials, Version 29). Data are presented as mean ± SD or *n* (%). To examine the differences in participant characteristics between at‐risk drinkers and low‐risk drinkers, a Chi‐square test was conducted for categorical variables and an independent *t*‐test was conducted for continuous variables. For continuous variables that were not normally distributed, the Mann–Whitney *U* test was conducted. To control for a potential effect of sex and use of antihypertensive medications, a univariate 2 × 2 × 2 ANOVA model was used for each outcome to test the three‐way interaction among sex (male vs. female), the use of antihypertensive medications (yes vs. no), and drinking group (at‐risk vs. low‐risk drinkers). When no significant three‐way interaction was observed, a sex by group two‐way univariate ANOVA model and a medication use by group two‐way univariate ANOVA model were examined in all participants for each outcome, followed by pairwise comparisons using Bonferroni tests to examine the differences between at‐risk drinkers and low‐risk drinkers. When indicated, the main effect of drinking group was examined. Pearson correlations were performed to identify any significant bivariate associations among USAUDIT‐C scores, central BP, wave reflection indices, arterial stiffness, and other potential confounding factors. Multiple linear regression models were conducted with central BP as the dependent variable and USAUDIT‐C scores as the primary predictor, sequentially adjusted for variables selected based on the results of bivariate associations. Multicollinearity was assessed using collinearity diagnostics. Alpha level was set as 0.05.

## 3 RESULTS

### 3.1 Participant characteristics and alcohol use

Compared with low‐risk drinkers, at‐risk drinkers had a higher USAUDIT‐C score, a higher level of PEth, a higher weekly amount of alcohol consumption, and more frequent binge alcohol use (*p* ≤ 0.001; Table 1). No differences between groups were found in age, BMI, and other measures of physical activity, sleep, diet, depression, and anxiety (*p* ≥ 0.13; Table 1). A total of 28 participants were on antihypertensive medications and the most commonly used antihypertensive medications were angiotensin receptor blockers (*n* = 10), followed by angiotensin‐converting enzyme inhibitors (*n* = 8) and beta‐blockers (*n* = 8; Table S1).

**TABLE 1:** Participant characteristics in midlife adult low‐risk drinkers and at‐risk drinkers.

	Low‐risk drinkers	At‐risk drinkers	*p* Value
	(*n* = 50)	(*n* = 29)	
Age (years)	58 ± 5	57 ± 4	0.43
Men (*n*)	21	17	0.15
Race			0.74
Native American (*n*)	1 (2)	0 (0)	
Asian (*n*)	4 (8)	2 (7)	
African American (*n*)	4 (8)	1 (3)	
Caucasian (*n*)	40 (80)	26 (90)	
More than one (*n*)	1 (2)	0 (0)	
Ethnicity			
Hispanic (*n*)	7 (14)	3 (10)	0.64
Antihypertensive medications (*n*)	15 (30)	13 (45)	0.18
Antilipid medications (*n*)	11 (22)	10 (34)	0.23
Antidepressants (*n*)	6 (12)	5 (17)	0.52
BMI (kg/m^2^)	27.3 ± 4.5	27.3 ± 3.6	0.99
Alcohol consumption			
USAUDIT‐C	3.5 ± 2.4	10.8 ± 2.7	<0.001
Average drinks/week	3 ± 4	18 ± 12	<0.001
Average drinking days/week	2 ± 2	5 ± 2	<0.001
Binge drinking days/week	0.1 ± 0.2	1.6 ± 1.8	<0.001
DBS PEth (ng/mL)	12 ± 32	115 ± 179	<0.001
MVPA (min/day)	27.6 ± 26.2	25.2 ± 26.8	0.35
Daily steps/day^a^	7408 ± 3517	6861 ± 3614	0.26
PSQI	5 ± 2	5 ± 3	0.43
Diet Habit Survey Score	153 ± 32	152 ± 27	0.22
PHQ‐8 Score^b^	2.7 ± 3.0	2.1 ± 1.9	0.13
STAI‐S Score^c^	27.4 ± 6.9	28.9 ± 9.3	0.22
STAI‐T Score^c^	30.7 ± 8.4	31.4 ± 6.6	0.37

*Note*: Data are mean ± SD or *n* (%). *p* Values are derived from the independent *t*‐test, Independent‐Samples Mann–Whitney *U* test if data are non‐normal distributed, or chi‐square. *p* < 0.05 indicates statistically significant and is highlighted in bold.

Abbreviations: BMI, body mass index; DBS PEth, dried blood spot phosphatidylethanol; MVPA, moderate to vigorous physical activity; PHQ‐8, eight‐item Personal Health Questionnaire Depression Scale; PSQI, Pittsburgh Sleep Quality Index; STAI‐S, the State–Trait Anxiety Inventory for state anxiety; STAI‐T, the State–Trait Anxiety Inventory for trail anxiety; USAUDIT‐C, the sum of the score from the first three questions in the US Alcohol Use Disorders Identification Test.

^a^ *n* = 50/27.

^b^ *n* = 46/25.

^c^ *n* = 47/25.

### 3.2 Effects of at‐risk drinking on central blood pressure, aortic wave reflection, and aortic stiffness

**FIGURE 1:**
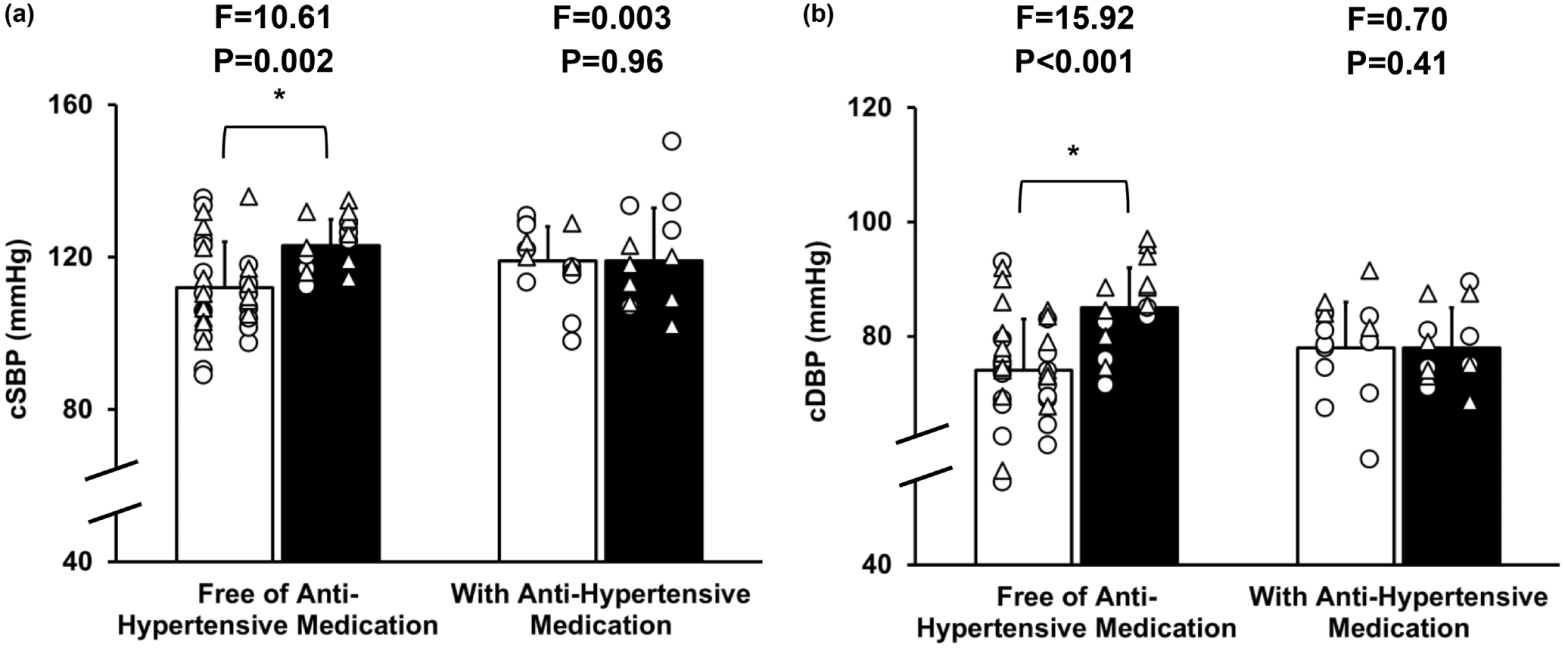
Comparisons between midlife low‐risk drinkers (white bar) and at‐risk drinkers (black bar), separated by antihypertensive medication use. (a) central systolic blood pressure (cSBP) and (b) central diastolic blood pressure (cDBP). White bars indicate group mean ± SD for low‐risk drinkers, black bars indicate group mean ± SD for at‐risk drinkers, open triangles (Δ) indicate individual data for men, and open circles (○) indicate individual data for women. *Indicates significant difference between low‐risk drinkers and at‐risk drinkers based on the pairwise comparisons analysis with Bonferroni correction from a univariate sex × medication use × drinking group ANOVA model.

There were no significant three‐way interactions among sex, use of antihypertensive medications, and drinking group (*p* ≥ 0.11) and no significant sex by group interactions (*p* ≥ 0.10; Table S2) for all outcomes. A significant interaction between use of antihypertensive medications and drinking group was observed for central BP (*p* ≤ 0.047), but not for other outcomes (*p* ≥ 0.25, Table S2). In participants free of antihypertensive medications, at‐risk drinkers had a higher level of central systolic and diastolic BP by 11 mmHg and 10 mmHg, respectively (*p* ≤ 0.002), while no difference in central BP was found between low‐risk drinkers and at‐risk drinkers who were on antihypertensive medications (*p* ≥ 0.41; Figure 1a,b). No main effects of drinking group were observed in central PP, HR, wave reflection indices, cfPWV, and cfPWV normalized to MAP (*p* ≥ 0.16; Table 2 and Table S2).

**TABLE 2:** Central blood pressure, wave reflection, and arterial stiffness in midlife adult low‐risk drinkers and at‐risk drinkers.

	Low‐risk drinkers	At‐risk drinkers	*p* Value
	(*n* = 50)	(*n* = 29)	
Pulse pressure (mmHg)	39 ± 7	40 ± 8	0.31
Heart rate (bpm)	60 ± 8	60 ± 9	0.52
Augmentation index (%)	33.8 ± 10.5	33.6 ± 9.3	0.43
Augmentation pressure (mmHg)	13 ± 5	14 ± 5	0.25
Reflection magnitude (%)	66.0 ± 9.2	65.5 ± 9.3	0.45
Forward pressure waves (mmHg)	25 ± 4	27 ± 5	0.37
Reflected pressure waves (mmHg)	17 ± 3	17 ± 4	0.18
cfPWV (m/sec)	7.8 ± 1.4	8.5 ± 1.5	0.16
cfPWV/MAP (m/sec•mmHg^−1^)	0.09 ± 0.01	0.09 ± 0.02	0.82

*Note*: Data are mean ± SD. *p* values are for the main effect of drinking group derived from a univariate 2 × 2 × 2 ANOVA model. *p* < 0.05 indicates statistical significance.

Abbreviations: cfPWV, carotid‐to‐femoral pulse wave velocity; cfPWV/MAP, carotid‐to‐femoral pulse wave velocity divided by mean arterial pressure.

**FIGURE 2:**
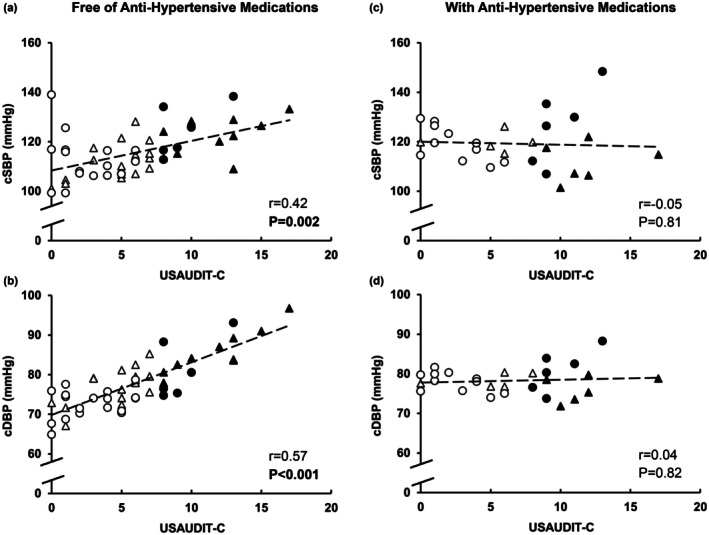
Pearson correlation between USAUDIT‐C and central systolic and diastolic blood pressure (cSBP and cDBP) in midlife adults free of antihypertensive medications (*n* = 51) and those taking antihypertensive medications (*n* = 28). Filled triangles (▲) indicate men at‐risk drinkers, open triangles (Δ) indicate men low‐risk drinkers, filled circles (●) indicate women at‐risk drinkers, and open circles (○) indicate women low‐risk drinkers. USAUDIT‐C is the sum of scores from the first three questions of the U.S. Alcohol Use Disorders Identification Test (USAUDIT).

Among midlife adults free of antihypertensive medications, higher USAUDIT‐C scores were associated with higher levels of central systolic BP (Figure 2a) and diastolic BP (Figure 2b). In contrast, among participants taking antihypertensive medications, USAUDIT‐C scores were not significantly associated with either central systolic BP (Figure 2c) or central diastolic BP (Figure 2d). In all participants, there were no associations between USAUDIT‐C score and measures of wave reflection (*p* ≥ 0.07; Table S3). While there was a positive association between USAUDIT‐C score and cfPWV (*r* = 0.23, *p* = 0.045), no association was found between USAUDIT‐C score and cfPWV normalized to MAP (*r* = 0.02, *p* = 0.89). In all participants (similar results regardless of antihypertension medication use; Table S3), a higher level of central systolic BP was correlated with a higher level of AP (*r* = 0.45, *p* < 0.001), a higher level of Pf (*r* = 0.70, *p* < 0.001), a higher level of Pb (*r* = 0.66, *p* < 0.001), and cfPWV (*r* = 0.61, *p* < 0.001), while a higher level of central diastolic BP was correlated with a higher level of Pf (*r* = 0.30, *p* = 0.008) and a higher level of cfPWV (*r* = 0.48, *p* < 0.001). Higher central BP levels were correlated with higher BMI levels (systolic: *r* = 0.26, *p* = 0.02 and diastolic: *r* = 0.13 and *p* = 0.24) and lower dietary habit survey scores (systolic: *r* = −0.36 and diastolic: *r* = −0.38, *p* < 0.001 for both), but unrelated to age, physical activity, sleep, and measures of mental health (*p* ≥ 0.11). After adjustment for potential confounders, higher USAUDIT‐C scores remained independently associated with elevated central systolic and diastolic BP among midlife adults free of antihypertensive medications (Table 3).

**TABLE 3:** Associations between USAUDIT‐C scores and central blood pressure in midlife adults.

		Free of antihypertensive medications (*n* = 51)	With antihypertensive medications (*n* = 28)
Central SBP	Model 1 (no adjustment)	1.19 (0.46–1.93) ***p* = 0.002**	−0.12 (−1.14–0.90) *p* = 0.81
	Model 2 (adjusted for AP, Pf, Pb, and cfPWV)	0.98 (0.42–1.54) ***p* < 0.001**	−0.01 (−0.57–0.55) *p* = 0.99
	Model 3 (adjusted for AP, Pf, Pb, cfPWV, and DHS score)	0.97 (0.44–3.82) ***p* < 0.001**	−0.05 (−0.60–0.49) *p* = 0.84
	Model 4 (adjusted for AP, Pf, Pb, cfPWV, DHS score, and BMI)	0.96 (0.43–1.50) ***p* < 0.001**	−0.11 (−0.65–0.42) *p* = 0.66
Central DBP	Model 1 (no adjustment)	1.33 (0.78–1.88) ***p* < 0.001**	0.07 (−0.58–0.72) *p* = 0.82
	Model 2 (adjusted for Pf and cfPWV)	1.06 (0.51–1.60) ***p* < 0.001**	0.10 (−0.51–0.70) *p* = 0.75
	Model 3 (adjusted for Pf, cfPWV, and DHS score)	1.06 (0.54–1.57) ***p* < 0.001**	0.08 (−0.54–0.69) *p* = 0.80

*Note*: Data are unstandardized B (95% confidence interval) derived from multiple linear regression models. USAUDIT‐C is the sum of scores from the first three questions of the U.S. Alcohol Use Disorders Identification Test (USAUDIT). *p* < 0.05 indicates statistically significant and is highlighted in bold.

Abbreviations: AP, augmentation index; BMI, body mass index; cfPWV, carotid‐to‐femoral pulse wave velocity; DHS, dietary habit survey; Pb, backward pressure waves; Pf, forward pressure waves; SBP, systolic blood pressure.

## 4 DISCUSSION

This is the first study to examine the effect of at‐risk drinking on central hemodynamics and arterial stiffness in midlife men and postmenopausal women who were nonsmokers and free of CVD and other major clinical diseases. We demonstrated that, regardless of sex, among participants free of antihypertensive medications, at‐risk drinkers had a higher level of central systolic and diastolic BP compared with low‐risk drinkers, while no between‐group differences in central BP were noted among treated participants. Regardless of sex and antihypertensive medication use, no differences between at‐risk drinkers and low‐risk drinkers were found in aortic wave reflection characteristics and stiffness. Among midlife adults free of antihypertensive medications, higher USAUDIT‐C scores remained independently associated with elevated central systolic and diastolic BP, after adjustment for aortic wave reflection and arterial stiffness.

Any amount of alcohol consumption has been linked to an increased risk of hypertension (Jones et al., 2025; Piano et al., 2025). A recent meta‐analysis of ~20,000 participants reported that regardless of sex, there was a positive, dose–response association between the amount of alcohol consumption at baseline and the level of BP after a median follow‐up of 5.3 years (Di Federico et al., 2023). Another meta‐analysis of >600,000 participants also reported a positive association between alcohol consumption above 1 drink/day and the risk of new‐onset hypertension (Cecchini et al., 2024). However, these studies targeted peripheral BP and the effect of alcohol consumption on central BP is less clear. We demonstrated that regardless of sex, in midlife adults free of antihypertensive medications, at‐risk drinkers had an increased level of central systolic BP by ~11 mmHg compared with low‐risk drinkers. This is clinically important, as a previous systemic review and meta‐analysis reported that for every 10‐mmHg increase in central systolic BP, there was an 8.8% increased risk of total cardiovascular events (Vlachopoulos, Aznaouridis, O'Rourke, et al., 2010). Moreover, another longitudinal study of 8710 participants (mean age: 50.1) with a median follow‐up of 6.4 years reported that compared with peripheral systolic BP, central systolic BP was more predictive of CVD events in participants with elevated CVD risks (Dong et al., 2020). We also demonstrated that in midlife adults free of antihypertensive medications, a higher USAUDIT‐C score was an independent predictor of a higher level of central BP. These findings, along with our previous findings in ambulatory BP measurements (Chang et al., 2025), suggest that a higher amount of weekly alcohol consumption and/or more occasions of binge drinking are associated with a higher BP profile, and midlife adults should limit alcohol consumption within the recommended levels to avoid increased risks of CVD.

Central BP is influenced by aortic stiffness and wave reflection. While there is growing evidence that at‐risk drinking is associated with increased aortic stiffness (Hwang et al., 2022), less is known regarding the effect of at‐risk drinking on aortic wave reflection and the evidence is limited to AIx, an index calculated as the percentage of central BP resulting from wave reflection. Findings from previous studies (Basdeki et al., 2021; Mahmud & Feely, 2002; Sierksma et al., 2004) suggest that while there is no association between alcohol consumption and AIx in women, at‐risk drinking is associated with an increased AIx in men. Compared to these studies, we controlled for smoking, several disease conditions, and the use of antihypertensive medications, and most importantly, provided a more detailed assessment of wave reflection. Our findings suggest that at‐risk drinking is not associated with wave reflection characteristics, including AIx, AP, the height of Pb, and RM, regardless of sex and use of antihypertensive medications. In agreement with our findings in aortic wave reflection characteristics, we found that there was no difference between low‐risk and at‐risk drinkers in cfPWV and cfPWV normalized by MAP. In addition, the association between USAUDIT‐C score and cfPWV became insignificant when cfPWV was normalized to MAP. These findings suggest that in midlife adults, aortic stiffening associated with at‐risk drinking may be attributable to an increase in BP. Arterial stiffness is BP dependent. Under the state of high BP or stretching of the arterial wall, the pressure load shifts to the stiffer components (such as collagen fibers), causing an increase in arterial stiffness, without a real structural change in the arterial wall. On the other hand, long‐term high BP can cause structural changes in the arterial wall, including elastin degradation and thus increased aortic stiffness. In addition to aortic stiffness, aortic wave reflection is determined by several other factors, including arterial stiffness in other parts of the arterial tree, the location of the impedance mismatch (vessel bifurcation or movement to a stiffer vessel), heart rate, and aortic diameter (Mitchell et al., 2003). To ultimately understand the time course of changes in central hemodynamics and arterial stiffness associated with alcohol use, longitudinal studies are warranted.

Our regression analysis results suggest that in midlife adults free of antihypertensive medications, the increase in central BP associated with at‐risk drinking is independent of aortic wave reflection and aortic stiffness. These findings suggest that the pressor effects of alcohol consumption in this age group are related to mechanisms other than enhanced aortic wave reflection and aortic stiffening. Blood pressure is determined by cardiac output (the product of heart rate and stroke volume) and total vascular resistance. In the same cohort of midlife adults, we previously reported no difference in resting heart rate and measures of heart rate variability between at‐risk drinkers and low‐risk drinkers (Chang et al., 2025). We also reported no difference in 24‐h, daytime, and nighttime urinary catecholamines between at‐risk drinkers and low‐risk drinkers (Chang et al., 2025). These findings suggest that the balance between cardiac parasympathetic and sympathetic tone at rest and catecholaminergic tone are less likely to be contributing factors of increased BP associated with at‐risk drinking. Fluid intake, such as the consumption of alcoholic beverages, increases blood volume, which stretches the atria, causing the secretion of atrial natriuretic peptide (ANP). Using data from the HyperGEN study, a cross‐sectional analysis of 1345 participants reported that higher amounts of weekly alcohol consumption were associated with higher plasma levels of ANP, even after controlling for end‐diastolic volume (Djousse et al., 2006). Several animal and human studies also reported that one‐time alcohol consumption increased ANP compared to a volume‐matched no‐alcoholic fluid condition (Colantonio et al., 1991; Gianoulakis et al., 1997). These findings suggest an increase in ANP associated with alcohol use may be independent of volume load and act to overcome the pressor effects of alcohol use, such as via diuresis, vasodilation, and/or the inhibition of the renin‐angiotensin‐aldosterone system (RAAS). Indeed, the long‐term pressor effect of alcohol use is potentially caused by the activation of RAAS (Da Silva et al., 2013), which can contribute to not only fluid retention, but also vasoconstriction, and thus an increased total vascular resistance and elevated BP. We postulated that medications that interfere with RAAS, such as angiotensin‐converting enzyme inhibitors and angiotensin receptor blockers, potentially contribute to the lack of differences in BP between at‐risk drinkers and low‐risk drinkers in midlife adults taking antihypertensive medications in our study. Future studies are needed to investigate the interaction between ANP and RSSA on central BP.

To our knowledge, this is the first study to investigate the effect of alcohol consumption on central hemodynamics in midlife men and postmenopausal women (50–64 years), who were nonsmokers and free of major clinical diseases. The inclusion and exclusion criteria may have contributed to a small sample size in this study. However, we included a relatively homogenous study sample to isolate the effect of alcohol use on central hemodynamics knowing that many factors would confound our results. We also measured several other potential confounding factors and found no differences in physical activity, diet, sleep, depression, and anxiety between low‐risk and at‐risk drinkers. In addition, we conducted statistical analyses to examine the interactions among sex, use of antihypertensive medications, and at‐risk drinking. Therefore, our findings were controlled for the abovementioned factors. We categorized drinking groups following the current guidelines specific to sex for Americans using the standardized questionnaire (USAUDIT‐C). These methodologies ensure the reproducibility of our study. We also used different tools to understand alcohol use over different timeframes, including an alcohol use biomarker (PEth), and confirmed that at‐risk drinkers had a higher amount and/or frequency of alcohol use compared to low‐risk drinkers.

## 5 CONCLUSIONS

Our findings suggest that in midlife adults, free of antihypertensive medications, drinking over the daily and weekly recommended limits or at‐risk drinking is associated with an increase in central BP. The increase in central BP associated with at‐risk drinking may be related to mechanisms other than enhanced aortic wave reflection and aortic stiffness.

## AUTHOR CONTRIBUTIONS

K‐Y.C., Z.L., R.M.B., S.A.P., and C‐L.H. conceived and designed research; K‐Y.C., Z.L., H.N., B.H., M.N., J.K., and C‐L.H. performed experiments; K‐Y.C., H.N., M.N., J.O.K., and C‐L.H. analyzed data; K‐Y.C., Z.L., H.N., B.H., J.O.K., Y.K., R.M.B., S.A.P., and C‐L.H. interpreted results of experiments; K‐Y.C., H.N., M.N., and C‐L.H. prepared figures; K‐Y.C., H.N., M.N., Y.K., and C‐L.H. drafted manuscript. K‐Y.C., Z.L., H.N., B.H., J.K., Y.K., R.M.B., S.A.P., and C‐L.H. edited and revised manuscript; K‐Y.C., Z.L., H.N., B.H., M.N., J.O.K., Y.K., R.M.B., S.A.P., and C‐L.H. approved final version of manuscript.

## FUNDING INFORMATION

This work is supported by the National Institute on Alcohol Abuse and Alcoholism (K99/R00 AA028537 to PI: Hwang and AA028537‐Supplement to John O. Kolade).

## ETHICS STATEMENT

This study was reviewed and approved by the Institutional Review Boards of the University of Illinois at Chicago (IRB# 2020‐0390) and the University of Texas at Arlington (IRB#2022‐0189).

## Supporting information


Tables S1–S3.

## Data Availability

Data can be made available upon reasonable request to the corresponding author. Part of the data, with the permission from research participants, will be shared on the National Institute on Alcohol Abuse and Alcoholism Data Archive (NIAAADA).

## References

[phy270717-bib-0001] Babor, T. F. , Higgins‐Biddle, J. C. , Dauser, D. , Burleson, J. A. , Zarkin, G. A. , & Bray, J. (2006). Brief interventions for at‐risk drinking: Patient outcomes and cost‐effectiveness in managed care organizations. Alcohol and Alcoholism, 41(6), 624–631. 10.1093/alcalc/agl078 17035245

[phy270717-bib-0002] Basdeki, E. D. , Tsirimiagkou, C. , Argyris, A. , Moschonis, G. , Sfikakis, P. , Protogerou, A. D. , & Karatzi, K. (2021). Moderately increased alcohol consumption is associated with higher pressure wave reflections and blood pressure in men. Nutrition, Metabolism, and Cardiovascular Diseases, 31(1), 85–94. 10.1016/j.numecd.2020.08.013 33500112

[phy270717-bib-0003] Ben‐Shlomo, Y. , Spears, M. , Boustred, C. , May, M. , Anderson, S. G. , Benjamin, E. J. , Boutouyrie, P. , Cameron, J. , Chen, C. H. , Cruickshank, J. K. , Hwang, S. J. , Lakatta, E. G. , Laurent, S. , Maldonado, J. , Mitchell, G. F. , Najjar, S. S. , Newman, A. B. , Ohishi, M. , Pannier, B. , … Wilkinson, I. B. (2014). Aortic pulse wave velocity improves cardiovascular event prediction: An individual participant meta‐analysis of prospective observational data from 17,635 subjects. Journal of the American College of Cardiology, 63(7), 636–646. 10.1016/j.jacc.2013.09.063 24239664 PMC4401072

[phy270717-bib-0004] Cecchini, M. , Filippini, T. , Whelton, P. K. , Iamandii, I. , di Federico, S. , Boriani, G. , & Vinceti, M. (2024). Alcohol intake and risk of hypertension: A systematic review and dose‐response meta‐analysis of nonexperimental cohort studies. Hypertension, 81(8), 1701–1715. 10.1161/HYPERTENSIONAHA.124.22703 38864208 PMC11251509

[phy270717-bib-0005] Chang, K. Y. , Haun, T. , Liu, Z. , Chang, K.‐. Y. , Gil, A. , Taherzadeh, Z. , Fadel, P. J. , Phillips, S. A. , Piano, M. R. , & Hwang, C.‐. L. (2025). Effects of at‐risk alcohol use on nighttime blood pressure, urinary catecholamines, and sleep quality in midlife adults. Alcoholism, Clinical and Experimental Research, 49, 843–853. 10.1111/acer.70021 PMC12912107 40059037

[phy270717-bib-0006] Colantonio, D. , Casale, R. , Desiati, P. , De Michele, G. , Mammarella, M. , & Pasqualetti, P. (1991). A possible role of atrial natriuretic peptide in ethanol‐induced acute diuresis. Life Sciences, 48(7), 635–642. 10.1016/0024-3205(91)90538-m 1824957

[phy270717-bib-0007] Da Silva, A. L. , Ruginsk, S. G. , Uchoa, E. T. , Crestani, C. C. , Scopinho, A. A. , Correa, F. M. , De Martinis, B. S. , Elias, L. L. , Resstel, L. B. , & Antunes‐Rodrigues, J. (2013). Time‐course of neuroendocrine changes and its correlation with hypertension induced by ethanol consumption. Alcohol and Alcoholism, 48(4), 495–504. 10.1093/alcalc/agt040 23733506

[phy270717-bib-0008] Di Federico, S. , Filippini, T. , Whelton, P. K. , Cecchini, M. , Iamandii, I. , Boriani, G. , & Vinceti, M. (2023). Alcohol intake and blood pressure levels: A dose‐response meta‐analysis of nonexperimental cohort studies. Hypertension, 80(10), 1961–1969. 10.1161/HYPERTENSIONAHA.123.21224 37522179 PMC10510850

[phy270717-bib-0009] Dietary Guidelines for Americans . (2020). 2020–2025 Dietary Guidelines for Americans 49.

[phy270717-bib-0010] Djousse, L. , Hunt, S. C. , Eckfeldt, J. H. , Arnett, D. K. , Province, M. A. , & Ellison, R. C. (2006). Alcohol consumption and plasma atrial natriuretic peptide (from the HyperGEN study). The American Journal of Cardiology, 98(5), 628–632. 10.1016/j.amjcard.2006.03.041 16923450

[phy270717-bib-0011] Dong, Y. , Jiang, L. , Wang, X. , Chen, Z. , Zhang, L. , Zhang, Z. , Zheng, C. , Kang, Y. , Wang, Z. , Cao, H. , Wang, X. , Fang, T. , Han, X. , Li, Z. , Tian, Y. , Dong, L. , Sun, F. , Yuan, F. , Zhou, X. , … Dai, C. (2020). Central rather than brachial pressures are stronger predictors of cardiovascular outcomes: A longitudinal prospective study in a Chinese population. Journal of Clinical Hypertension (Greenwich, Conn.), 22(4), 623–630. 10.1111/jch.13838 32153115 PMC8029759

[phy270717-bib-0012] Esselink, A. , Bovens, R. , de Van Mheen, D. H. M. , Gesthuizen, M. J. W. , & Mathijssen, J. J. P. (2023). Towards a new definition of the typical day in the alcohol use disorder identification test‐consumption. European Addiction Research, 29(4), 264–271. 10.1159/000530823 37311446 PMC10614262

[phy270717-bib-0013] Gianoulakis, C. , Guillaume, P. , Thavundayil, J. , & Gutkowska, J. (1997). Increased plasma atrial natriuretic peptide after ingestion of low doses of ethanol in humans. Alcoholism, Clinical and Experimental Research, 21(1), 162–170. 9046389

[phy270717-bib-0014] Grucza, R. A. , Sher, K. J. , Kerr, W. C. , Krauss, M. J. , Lui, C. K. , McDowell, Y. E. , Hartz, S. , Virdi, G. , & Bierut, L. J. (2018). Trends in adult alcohol use and binge drinking in the early 21st‐century United States: A meta‐analysis of 6 National Survey Series. Alcoholism, Clinical and Experimental Research, 42(10), 1939–1950. 10.1111/acer.13859 30080258 PMC6364977

[phy270717-bib-0015] Hashimoto, J. (2017). Central hemodynamics for management of arteriosclerotic diseases. Journal of Atherosclerosis and Thrombosis, 24(8), 765–778. 10.5551/jat.40717 28603219 PMC5556183

[phy270717-bib-0016] Hwang, C. L. , Muchira, J. , Hibner, B. A. , Phillips, S. A. , & Piano, M. R. (2022). Alcohol consumption: A new risk factor for arterial stiffness? Cardiovascular Toxicology, 22(3), 236–245. 10.1007/s12012-022-09728-8 35195845 PMC8863568

[phy270717-bib-0017] Hwang, C. L. , Piano, M. R. , & Phillips, S. A. (2021). The effects of alcohol consumption on flow‐mediated dilation in humans: A systematic review. Physiological Reports, 9(10), e14872. 10.14814/phy2.14872 34042304 PMC8157766

[phy270717-bib-0018] Hwang, M. H. , Yoo, J. K. , Kim, H. K. , Hwang, C. L. , Mackay, K. , Hemstreet, O. , Nichols, W. W. , & Christou, D. D. (2014). Validity and reliability of aortic pulse wave velocity and augmentation index determined by the new cuff‐based SphygmoCor Xcel. Journal of Human Hypertension, 28(8), 475–481. 10.1038/jhh.2013.144 24430704

[phy270717-bib-0019] Jones, D. W. , Ferdinand, K. C. , Taler, S. J. , Johnson, H. M. , Shimbo, D. , Abdalla, M. , Altieri, M. M. , Bansal, N. , Bello, N. A. , Bress, A. P. , Carter, J. , Cohen, J. B. , Collins, K. J. , Commodore‐Mensah, Y. , Davis, L. L. , Egan, B. , Khan, S. S. , Lloyd‐Jones, D. M. , Melnyk, B. M. , … Williamson, J. D. (2025). 2025 AHA/ACC/AANP/AAPA/ABC/ACCP/ACPM/AGS/AMA/ASPC/NMA/PCNA/SGIM guideline for the prevention, detection, evaluation and Management of High Blood Pressure in adults: A report of the American College of Cardiology/American Heart Association joint committee on clinical practice guidelines. Circulation, 152, e114–e218. 10.1161/CIR.0000000000001356 40811497

[phy270717-bib-0020] Mahmud, A. , & Feely, J. (2002). Divergent effect of acute and chronic alcohol on arterial stiffness. American Journal of Hypertension, 15(3), 240–243. 10.1016/s0895-7061(01)02315-9 11939614

[phy270717-bib-1001] Martin, S. S. , Aday, A. W. , Allen, N. B. , Zaid, A., I , Anderson, C. A. M. , Arora, P. , Avery, C. L. , Baker‐Smith, C. M. , Bansal, N. , Beaton, A. Z. , Commodore‐Mensah, Y. , Currie, M. E. , Elkind, M. S. V. , Fan, W. , Generoso, G. , Gibbs, B. B. , Heard, D. G. , Hiremath, S. , Johansen, M. C. , … American Heart Association Council on Epidemiology and Prevention Statistics Committee and Stroke Statistics Committee . (2025). 2025 Heart disease and stroke statistics: A report of US and global data from the American Heart Association. Circulation, 151(8). 10.1161/CIR.0000000000001303 PMC12256702 39866113

[phy270717-bib-0021] Mitchell, G. F. , Lacourciere, Y. , Ouellet, J. P. , Izzo, J. L., Jr. , Neutel, J. , Kerwin, L. J. , Block, A. J. , & Pfeffer, M. A. (2003). Determinants of elevated pulse pressure in middle‐aged and older subjects with uncomplicated systolic hypertension: The role of proximal aortic diameter and the aortic pressure‐flow relationship. Circulation, 108(13), 1592–1598. 10.1161/01.CIR.0000093435.04334.1F 12975261

[phy270717-bib-0022] Mitchell, G. F. , Parise, H. , Benjamin, E. J. , Larson, M. G. , Keyes, M. J. , Vita, J. A. , Vasan, R. S. , & Levy, D. (2004). Changes in arterial stiffness and wave reflection with advancing age in healthy men and women: The Framingham heart study. Hypertension, 43(6), 1239–1245. 10.1161/01.HYP.0000128420.01881.aa 15123572

[phy270717-bib-0023] Piano, M. R. , Marcus, G. M. , Aycock, D. M. , Buckman, J. , Hwang, C. L. , Larsson, S. C. , Mukamal, K. J. , Roerecke, M. , & on behalf the American Heart Association Council on Lifestyle and Cardiometabolic Health; Council on Cardiovascular and Stroke Nursing; Council on Clinical Cardiology; and Stroke Council . (2025). Alcohol use and cardiovascular disease: A scientific statement from the American Heart Association. Circulation, 152, e7–e21. 10.1161/CIR.0000000000001341 40485439

[phy270717-bib-0024] Sierksma, A. , Lebrun, C. E. , van der Schouw, Y. T. , Grobbee, D. E. , Lamberts, S. W. , Hendriks, H. F. , & Bots, M. L. (2004). Alcohol consumption in relation to aortic stiffness and aortic wave reflections: A cross‐sectional study in healthy postmenopausal women. Arteriosclerosis, Thrombosis, and Vascular Biology, 24(2), 342–348. 10.1161/01.ATV.0000110784.52412.8f 14656732

[phy270717-bib-0026] Verhoog, S. , Dopmeijer, J. M. , de Jonge, J. M. , van der Heijde, C. M. , Vonk, P. , Bovens, R. H. L. M. , de Boer, M. R. , Hoekstra, T. , Kunst, A. E. , Wiers, R. W. , & Kuipers, M. A. G. (2020). The use of the alcohol use disorders identification test ‐ consumption as an indicator of hazardous alcohol use among university students. European Addiction Research, 26(1), 1–9. 10.1159/000503342 31563902 PMC6979415

[phy270717-bib-0027] Vlachopoulos, C. , Aznaouridis, K. , O'Rourke, M. F. , Safar, M. E. , Baou, K. , & Stefanadis, C. (2010). Prediction of cardiovascular events and all‐cause mortality with central haemodynamics: A systematic review and meta‐analysis. European Heart Journal, 31(15), 1865–1871. 10.1093/eurheartj/ehq024 20197424

[phy270717-bib-0028] Vlachopoulos, C. , Aznaouridis, K. , & Stefanadis, C. (2010). Prediction of cardiovascular events and all‐cause mortality with arterial stiffness: A systematic review and meta‐analysis. Journal of the American College of Cardiology, 55(13), 1318–1327. 10.1016/j.jacc.2009.10.061 20338492

