# Supplementary material for: Effects of at‐risk drinking on central hemodynamics and aortic stiffness in midlife adults
Source: Physiol Rep. 2026 Jan 11;14(1):e70717. doi: 10.14814/phy2.70717 (PMC12791031; doi:10.14814/phy2.70717)
Supplement: Supplementary file 1 — Tables S1–S3. [file PHY2-14-e70717-s001.pdf]

Supplemental Table 1. Classes of Anti-Hypertensive Medications in Mid-Life Adult Low-Risk Drinkers and At-Risk Drinkers

	Low-Risk Drinkers (n=15)	At-Risk Drinkers (n=13)	*P* Value
**CCBs (n)**	2 (13)	3 (23)	0.26
**ACEIs (n)**	3 (20)	5 (38)	0.11
**ARBs (n)**	6 (40)	4 (31)	0.82
**ARAs (n)**	1 (7)	0 (0)	0.44
**Diuretics (n)**	2 (13)	0 (0)	0.28
**Beta-Blockers (n)**	3 (20)	5 (38)	0.11

Data are n (%).

CCBs, calcium channel blockers; ACEIs, angiotensin-converting enzyme inhibitors; ARBs, angiotensin II receptor blockers; and ARAs, aldosterone receptor antagonists.

P values are derived from Chi-Square. P<0.05 indicates statistically significant and is highlighted in bold.

Supplemental Table 2. Summary of ANOVA models for Central Hemodynamics and Aortic Stiffness.

	Sex x Use of Anti-HTN Medications x Alcohol Group	Use of Anti-HTN Medications x Alcohol Group	Sex x Alcohol Group	Alcohol Group
**SBP**	2.66, 0.11	**4.10, 0.047**	2.76, 0.10	-
**DBP**	2.56, 0.11	**9.57, 0.003**	1.24, 0.27	-
**PP**	0.46, 0.50	0.04, 0.85	1.74, 0.19	1.03, 0.31
**HR**	0.21, 0.65	0.92, 0.34	0.08, 0.78	0.41, 0.52
**AIx**	<0.0005, >0.99	0.07, 0.79	<0.0005, >0.99	0.62, 0.43
**AP**	0.29, 0.59	0.12, 0.74	0.52, 0.47	1.33, 0.25
**RM**	0.37, 0.55	1.33, 0.25	0.19, 0.67	0.57, 0.45
**Pf**	1.28, 0.26	0.69, 0.41	2.77, 0.10	0.83, 0.37
**Pb**	0.48, 0.49	0.06, 0.82	1.41, 0.24	1.89, 0.18
**cfPWV**	<0.0005, >0.99	1.13, 0.29	0.55, 0.46	1.96, 0.16
**cfPWV/MAP**	1.51, 0.22	0.22, 0.64	0.01, 0.92	0.05, 0.82

Data are F values, P values derived from a univariate 2 × 2 × 2 ANOVA model for each outcome.

SBP, systolic blood pressure; DBP, diastolic blood pressure; PP, pulse pressure; AP, augmentation index; HR, heart rate; AIx, augmentation index; AP, augmentation pressure; RM, reflection magnitude; Pf, forward pressure waves; Pb, backward pressure waves; cfPWV, carotid-to-femoral pulse wave velocity; MAP, mean arterial pressure.

P<0.05 indicates statistically significant and is highlighted in bold.

Supplemental Table 3. Simple correlation between USAUDIT-C scores, Central Blood pressure, Aortic Wave Reflection, and Aortic Stiffness in Midlife-adults with and without anti-hypertensive medications.

	**USAUDIT-C scores**			**Central SBP**			**Central DBP**		
	**All (n=79)**	**Untreated (n=51)**	**Treated (n=28)**	**All (n=79)**	**Untreated (n=51)**	**Treated (n=28)**	**All (n=79)**	**Untreated (n=51)**	**Treated (n=28)**
**AIx**	-0.17	-0.23	-0.04	0.19	0.19	0.20	-0.06	-0.08	0.02
	0.14	0.11	0.82	0.10	0.18	0.32	0.63	0.57	0.91
**AP**	-0.15	-0.21	-0.06	0.45	0.39	0.56	-0.01	-0.05	0.07
	0.19	0.14	0.77	**<0.001**	**0.004**	**0.002**	0.93	0.76	0.72
**RM**	-0.21	-0.30	-0.08	0.08	0.03	0.18	0.08	-0.24	-0.16
	0.07	**0.03**	0.69	0.47	0.83	0.37	0.47	0.08	0.41
**Pf**	0.07	0.12	-0.02	0.70	0.66	0.75	0.30	0.28	0.36
	0.53	0.41	0.91	**<0.001**	**<0.001**	**<0.001**	**0.008**	**0.048**	0.06
**Pb**	-0.10	-0.14	-0.07	0.66	0.57	0.78	0.10	0.06	0.18
	0.39	0.34	0.74	**<0.001**	**<0.001**	**<0.001**	0.38	0.66	0.36
**cfPWV**	0.23	0.34	-0.03	0.61	0.63	0.54	0.48	0.51	0.42
	**0.045**	**0.01**	0.87	**<0.001**	**<0.001**	**0.003**	**<0.001**	**<0.001**	**0.03**
**cfPWV/MAP**	0.02	0.29	-0.05	0.09	0.10	-0.13	-0.10	-0.07	-0.20
	0.89	**0.04**	0.80	0.44	0.49	0.51	0.37	0.61	0.30

Data are r and p values derived from the Pearson correlation analysis.

USAUDIT-C is the sum of scores from the first three questions of the U.S. Alcohol Use Disorders Identification Test (USAUDIT). SBP, systolic blood pressure; DBP, diastolic blood pressure; PP, pulse pressure; AP, augmentation index; AIx, augmentation index; AP, augmentation pressure; RM, reflection magnitude; Pf, forward pressure waves; Pb, backward pressure waves; cfPWV, carotid-to-femoral pulse wave velocity; MAP, mean arterial pressure.

P<0.05 indicates statistically significant and is highlighted in bold.
